# Motor abilities in adults born with very low birthweight: A study of two birth cohorts from Finland and Norway

**DOI:** 10.1111/dmcn.15883

**Published:** 2024-02-18

**Authors:** Silje D. Benum, Kristina A. D. Aakvik, Anna P. M. Jørgensen, Laura Jussinniemi, Maarit Kulmala, Maria Vollsæter, Eero Kajantie, Kari Anne I. Evensen

**Affiliations:** ^1^ Department of Clinical and Molecular Medicine Norwegian University of Science and Technology Trondheim Norway; ^2^ Department of Neuromedicine and Movement Science Norwegian University of Science and Technology Trondheim Norway; ^3^ Clinical Medicine Research Unit Oulu University Hospital and University of Oulu Oulu Finland; ^4^ Public Health Unit, Finnish Institute for Health and Welfare Helsinki Finland; ^5^ Helsinki University Eye and Ear Hospital Helsinki Finland; ^6^ Department of Pediatrics Haukeland University Hospital Bergen Norway; ^7^ Department of Clinical Science University of Bergen Bergen Norway; ^8^ Children's Hospital, Helsinki University Hospital and University of Helsinki Helsinki Finland; ^9^ Department of Rehabilitation Science and Health Technology Oslo Metropolitan University Oslo Norway; ^10^ Children's Clinic, St. Olavs Hospital Trondheim University Hospital Trondheim Norway

## Abstract

**Aim:**

To compare overall, fine, and gross motor abilities in adults born preterm with very low birthweight (VLBW) and a control group of term‐born individuals.

**Method:**

In a joint assessment of the Helsinki Study of Very Low Birth Weight Adults and NTNU Low Birth Weight in a Lifetime Perspective study, data were collected with harmonized methods for 118 adults born preterm (gestational age < 37 weeks) with VLBW (≤1500 g) and 147 control individuals. The primary outcome was overall motor abilities; secondary outcomes were fine and gross motor abilities.

**Results:**

The Bruininks Motor Ability Test Short Form total score was 4.1 (95% confidence interval 2.7–6.0) points lower in adults born with VLBW than in the control group, adjusted for cohort, age, and sex. This was partly mediated by their shorter height. They also had lower scores for other fine and gross motor tests. Results were similar when participants with neurosensory impairment were excluded, and when we adjusted for additional covariates.

**Interpretation:**

Adults born preterm with VLBW had poorer overall, fine, and gross motor abilities than adults born at term. This indicates that substantial difficulties in motor function among individuals born preterm with VLBW persist into mid‐adulthood.

## Graphical abstract

In this study of two birth cohorts from Finland and Norway, we found reduced overall, fine, and gross motor abilities among adults born preterm with very low birthweight (VLBW ≤1500 g) at mean age 36 years compared with term‐born individuals. This indicates that substantial difficulties in motor function among individuals born preterm with VLBW persist into mid‐adulthood.

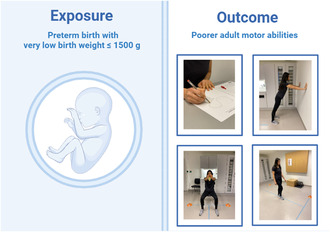

**Abbreviations**
BMAT: Bruininks Motor Ability Test
GP: Grooved Pegboard
HeSVA: Helsinki Study of Very Low Birth Weight Adults
HiMAT: High‐level mobility Assessment Tool
NSI: neurosensory impairment
NTNU LBW Life: NTNU Low Birth Weight in a Lifetime Perspective
NTNU: Norwegian University of Science and Technology
TMT‐5: Trail Making Test‐5
VLBW: very low birthweight

What this paper adds

- Adults born with very low birthweight had poorer motor abilities than term‐born individuals.
- This was evident in several motor tests of overall, fine, and gross motor abilities.
- Reduced motor speed was observed in several of the motor tests.

Motor difficulties have been consistently reported in children born preterm with very low birthweight (VLBW ≤ 1500 g).^1^, ^2^, ^3^ Even in the absence of cerebral palsy (CP), the prevalence of motor difficulties is up to sixfold higher in school‐aged children born very preterm or with VLBW than in term‐born children with normal birthweight.^2^ The motor difficulties are evident in manual dexterity, ball skills, and balance.^1^, ^3^

Childhood motor difficulties are associated with later mental health problems among adults born with extremely low birthweight (<1000 g)^4^ and decreasing self‐concept among adults born very preterm/VLBW.^5^ Furthermore, motor difficulties may interfere with social and daily living activities, physical activity, and academic achievement.^6^ Evidence is scarce about motor abilities in the adult population who were born VLBW, but a few studies indicate that motor difficulties persist into young adulthood. We have previously reported poorer overall motor skills and reduced motor speed on fine motor tasks in adults born with VLBW at 23 years compared with a control group.^7^ No studies have clinically assessed motor abilities of adults born with VLBW beyond this age,^1^ but a higher prevalence of self‐reported motor difficulties has been reported in adults born with extremely low birthweight than in control individuals at 36 years.^8^

In a joint study of two birth cohorts from Finland and Norway, our aim was to compare clinically assessed motor abilities of adults born with VLBW and a control group of term‐born individuals. We hypothesized that adults born with VLBW have more overall, fine, and gross motor difficulties than term‐born adults, especially on timed performances requiring motor speed.

## METHOD

### Study design

Data were collected with harmonized methods in a joint assessment of two longitudinal birth cohorts: the Helsinki Study of Very Low Birth Weight Adults (HeSVA) from Finland and the NTNU Low Birth Weight in a Lifetime Perspective (NTNU LBW Life) from Norway.^9^ HeSVA included infants with VLBW (birthweight < 1500 g) born between 1978 and 1985 who were discharged from the neonatal intensive care unit at the Children's Hospital in Helsinki.^10^ Control individuals born at term (gestational age ≥ 37 weeks) not small for gestational age (birthweight for gestational age ≥ −2 standard deviations [SD]) matched for age, sex, and birth hospital were recruited during a study visit in 2004 to 2005. The NTNU LBW Life study included infants born with VLBW (birthweight ≤ 1500 g) between 1986 and 1988 who were admitted to the neonatal intensive care unit at the University Hospital in Trondheim.^11^ Control individuals born at term (gestational age ≥ 37 weeks) not small for gestational age (birthweight for gestational age ≥ 10th centile), corrected for sex and parity, were recruited from a multicentre study enrolling pregnant females between 1986 and 1988. Assessments were performed between September 2019 and January 2021.

### Study groups

#### VLBW group

There were 188 individuals with VLBW from HeSVA and 83 from NTNU LBW Life (Figure S1) who were eligible. Of these, 175 from HeSVA and 72 from NTNU LBW Life were invited to participate, of whom 110 did not consent. Altogether, 118 individuals with VLBW, corresponding to 47.8% of those invited, had their motor abilities assessed.

#### Control group

There were 190 control individuals from HeSVA and 118 from NTNU LBW Life (Figure S1) who were eligible. Of these, 166 from HeSVA and 104 from NTNU LBW Life were invited to participate, of whom 112 did not consent. Altogether, 147 control individuals, corresponding to 54.4% of those invited, had their motor abilities assessed.

### Non*‐*participants

Background characteristics of participants and individuals who did not consent to participation, or who did not have their motor abilities assessed in the VLBW and control groups, are shown in Table 1. A detailed analysis of non‐participants was recently published and indicated no differences in neonatal characteristics between participants who attended the study and those who were invited but did not attend.^9^

**TABLE 1:** Background characteristics of participants and individuals who did not consent to participation or who did not have their motor abilities assessed in the VLBW and control groups.

	VLBW						Control					
	Participants			Non‐participants			Participants			Non‐participants		
	*n*			*n*			*n*			*n*		
Maternal age at delivery (years:months), mean (SD)	118	29:11	(4:8)	129	28:7	(4:10)	146	30:2	(4:11)	120	29:5	(4:2)
Gestational age (weeks), mean (SD)	118	29.5	(2.5)	129	28.8	(2.3)	147	40.0	(1.2)	123	40.1	(1.2)
Birthweight (g), mean (SD)	118	1174	(213)	129	1121	(223)	147	3645	(485)	123	3651	(434)
Birth head circumference (cm), mean (SD)	113	26.7	(2.1)	114	26.3	(2.1)	124	35.2	(1.2)	100	35.3	(1.3)
Apgar score after 1 minute, mean (SD)	97	6.3	(2.4)	103	5.6	(2.6)	123	8.8	(0.6)	99	8.9	(0.5)
Apgar score after 5 minutes, mean (SD)	78	7.8	(2.0)	85	7.3	(2.0)	63	9.7	(1.2)	44	9.9	(0.3)
Days of stay in NICU, mean (SD)	81	69.9	(43.6)	84	74.4	(31.9)	—	—	—	—	—	—
Days on ventilator, mean (SD)	115	7.4	(13.1)	128	11.3	(16.3)	—	—	—	—	—	—
Sepsis, *n* (%)	115	9	(7.8)	119	12	(10.1)	—	—	—	—	—	—
Interventricular haemorrhage, *n* (%)	92	15	(16.3)	98	21	(21.4)	—	—	—	—	—	—
Respiratory distress syndrome, *n* (%)	116	55	(47.4)	128	71	(55.5)	—	—	—	—	—	—
Bronchopulmonary dysplasia, *n* (%)	111	30	(27.0)	125	46	(36.8)	—	—	—	—	—	—
CP, *n* (%)	118	7	(5.9)	120	9	(7.5)	147	1	(0.7)	—	—	—
NSI, *n* (%)	118	10	(8.5)	122	23	(18.9)	144	1	(0.7)	120	2	(1.7)
Maternal education, *n* (%)	114			122			135			107		
Basic or less		31	(27.2)		35	(28.7)		33	(24.4)		29	(27.1)
Secondary		24	(21.1)		30	(24.6)		29	(21.5)		15	(14.0)
Lower‐level tertiary		35	(30.7)		45	(36.9)		39	(28.9)		32	(29.9)
Upper‐level tertiary		24	(21.1)		12	(9.8)		34	(25.2)		31	(29.0)
Paternal education, *n* (%)	111			119			134			106		
Unknown		1	(0.9)		0	(0.0)		0	(0.0)		0	(0.0)
Basic or less		33	(29.7)		39	(32.8)		32	(23.9)		31	(29.2)
Secondary		25	(22.5)		29	(24.4)		33	(24.6)		20	(18.9)
Lower‐level tertiary		28	(25.2)		34	(28.6)		28	(20.9)		28	(26.4)
Upper‐level tertiary		24	(21.6)		17	(14.3)		41	(30.6)		27	(25.5)

Abbreviations: CP, cerebral palsy; NICU, neonatal intensive care unit; NSI, neurosensory impairment; SD, standard deviation; VLBW, very low birthweight.

### Background and clinical characteristics

Maternal age at delivery, gestational age, birthweight, head circumference, Apgar score after 1 minute and 5 minutes, days of stay in the neonatal intensive care unit, days on ventilator, sepsis, presence of interventricular haemorrhage, respiratory distress syndrome, and bronchopulmonary dysplasia (supplemental oxygen ≥ 28 days) were retrieved from hospital records. CP was assessed by self‐report at the current follow‐up in HeSVA and diagnosed by a clinician at the 14‐year follow‐up or self‐reported at the current follow‐up in NTNU LBW Life. Neurosensory impairment (NSI) was defined as having CP, blindness, deafness or hearing aid, and/or intellectual disability defined by self‐report in young adulthood (HeSVA) or clinically assessed IQ less than 70 at 19 years, 14 years, or 5 years (NTNU LBW Life). Data on maternal and paternal education were collected in young adulthood (HeSVA) and at the 14‐ or 19‐year follow‐up (NTNU LBW Life), and categorized into education levels (basic or less, secondary, lower‐level tertiary, or upper‐level tertiary) based on the International Standard Classification of Education.^12^

At the current follow‐up, height was measured to the nearest 0.1 cm. Weight was measured to the nearest 0.1 kg using a Seca medical Body Composition Analyzer (Seca® mBCA 515, Hamburg, Germany) or a Seca electronical weight, and body mass index (kg/m^2^) was calculated. Body mass index was grouped in four categories (underweight, normal weight, overweight, and obese) according to the classification of the World Health Organization. Handedness was assessed by the Edinburgh Handedness Inventory Short Form, where participants reported their preferred hand for writing, throwing, using a toothbrush, and using a spoon, resulting in categorization into right‐, mixed‐, or left‐handedness.^13^ Data on participants' education were collected by self‐report and categorized into the education levels of lower secondary or lower (International Standard Classification of Education 1–2), intermediate (3–5), or lower tertiary or higher (6–8).

Clinical characteristics of adults with VLBW and control individuals are shown in Table 2.

**TABLE 2:** Clinical characteristics of the VLBW and control groups.

	VLBW (*n* = 118)		Control (*n* = 147)	
Female, *n* (%)	68	(57.6)	85	(57.8)
Age (years:months), mean (SD)	36:2	(3:2)	35:10	(3:2)
Height (cm), mean (SD)	168.2	(9.8)	173.6	(9.6)
Weight (kg), mean (SD)	73.3	(17.5)	77.4	(15.1)
BMI (kg/m^2^), mean (SD)	26.0	(6.0)	25.6	(4.4)
BMI classification, *n* (%)				
Underweight (BMI <18.5)	4	(3.4)	0	(0.0)
Normal weight (BMI 18.5–24.9)	56	(47.5)	75	(51.0)
Overweight (BMI 25.0–29.9)	31	(26.3)	50	(34.0)
Obesity (BMI ≥30.0)	27	(22.9)	22	(15.0)
Handedness, *n* (%)				
Right	98	(83.1)	130	(88.4)
Mixed	10	(8.5)	11	(7.5)
Left	10	(8.5)	6	(4.1)
Education, *n* (%)				
Lower secondary or lower (ISCED 1–2)	4	(3.4)	2	(1.4)
Intermediate (ISCED 3–5)	55	(46.6)	48	(32.7)
Lower tertiary or higher (ISCED 6–8)	59	(50.0)	97	(66.0)

Abbreviations: BMI, body mass index; ISCED, International Standard Classification of Education; SD, standard deviation; VLBW, very low birthweight.

### Outcome measures

The primary outcome was overall motor abilities assessed by the Bruininks Motor Ability Test (BMAT) Short Form. Secondary outcomes were fine motor abilities assessed by the Grooved Pegboard (GP) and the Trail Making Test‐5 (TMT‐5), and gross motor abilities assessed by the Revised High‐level Mobility Assessment Tool (HiMAT). Two trained study nurses performed motor assessments of the HeSVA cohort, and two trained physiotherapists and a medical research student performed assessments of the NTNU LBW Life cohort. The examiners performed an audit before and during data collection to evaluate assessments and procedures. Examiners were blinded to group adherence, medical history, clinical characteristics, and results from previous follow‐up.

#### Overall motor abilities

The BMAT Short Form is designed to assess overall motor abilities related to activities of daily living in adults aged 40 years and older.^14^ It includes 10 items grouped into five subtests. Raw scores are recorded and converted into point scores for each item and subtest according to the test manual: Fine Motor Integration (0–14 points), Manual Dexterity (0–18 points), Coordination (0–10 points), Balance and Mobility (0–9 points), and Strength and Flexibility (0–18 points). Skipping or terminating an item is scored zero. Point scores for each subtest add up to a total score which ranges from 0 to 69.

#### Fine motor abilities

The GP is a manipulative dexterity test for adults which requires the use of complex visual–motor coordination to place pegs into 25 keyhole‐shaped holes with randomly positioned slots.^15^ It has been used in several clinical populations.^15^ Time in seconds to complete the test was recorded for the dominant and non‐dominant hand.

The TMT‐5 is part of the Delis‐Kaplan Executive Function System which is designed to evaluate higher‐level cognitive function in children, adolescents, and adults.^16^ The TMT‐5 specifically measures motor speed by having the participant draw a line between 32 circles as fast as possible in a directed order. Time in seconds to complete the test was recorded.

#### Gross motor abilities

The HiMAT measures gross motor abilities in adolescents and adults; it was initially developed to assess high‐level mobility outcomes in adults with traumatic brain injury. It could also be applied to other neurological conditions.^17^ The Revised HiMAT includes eight items: walk, walk backward, walk on toes, walk over obstacle, run, skip, hop forward (more affected leg), and bound (less affected leg).^18^ Items are performed on a 20 m walkway marked by cones and the middle 10 m is timed for all items except for the bound item where distance in centimetres is measured. Failure, refusal, or inability to perform the item is scored zero. Raw item scores were converted into standardized scores from 0 to 4 according to the manual, adding up to a total score of 0 to 32 points.

### Ethical considerations

The project adhered to the Helsinki Declaration and was approved by the Ethics Committee IV of Helsinki University Hospital (HUS/1157) in Finland and the Regional Committee for Medical Research Ethics in Central‐Norway (23879). All methods were non‐invasive and entailed very low risk for injury or adverse events. Written informed consent was obtained from all participants to the research and to publication of results. At each university hospital, appointed doctors were medically responsible during the data collection. If there was a need for health services, participants were referred as appropriate.

### Statistical analyses

**FIGURE 1:**
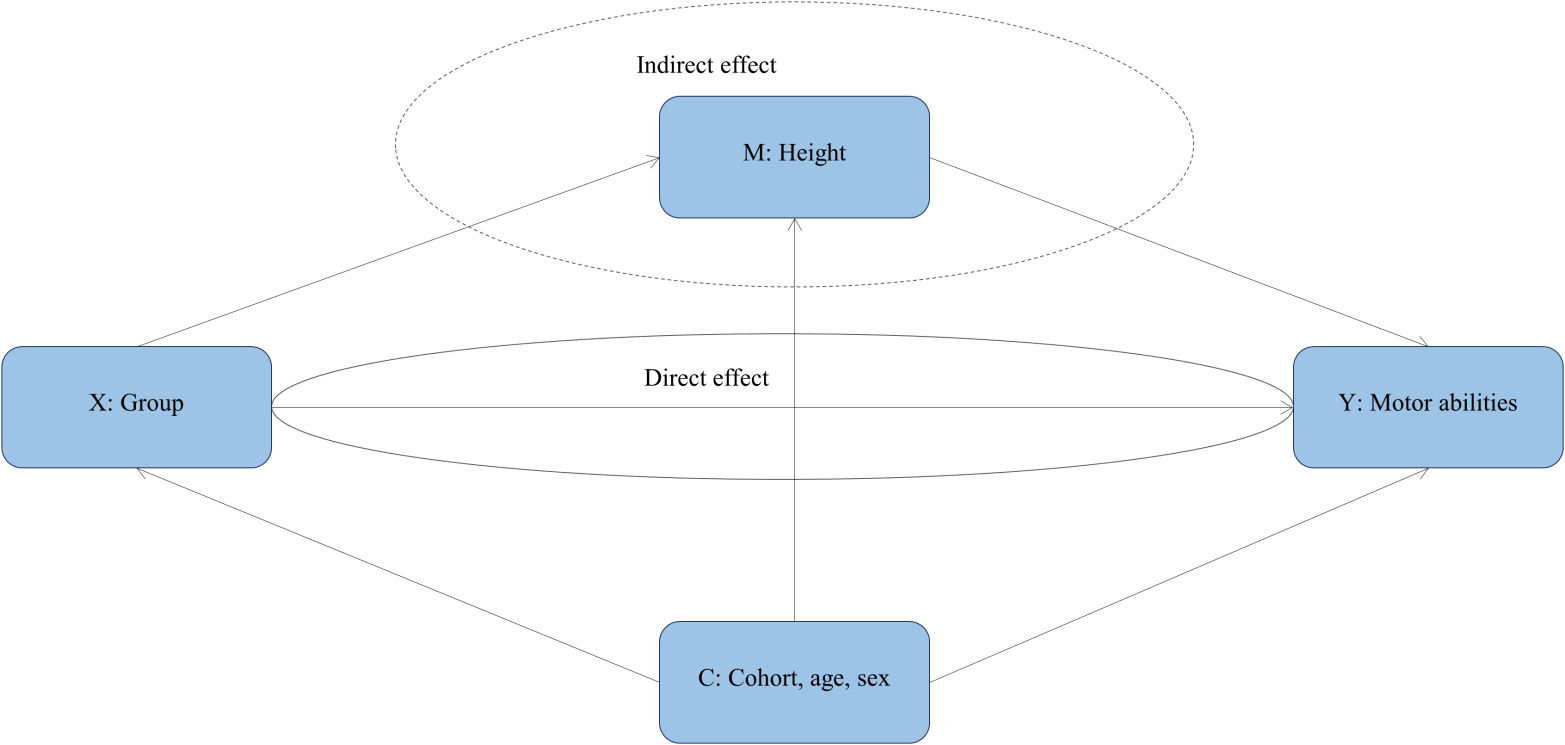
Relation between group (X), height (M), and motor abilities (Y) with confounders (C). Group: VLBW versus control. Motor abilities: Bruininks Motor Ability Test Short Form scores or Revised High‐level Mobility Assessment Tool scores. Abbreviation: VLBW, very low birthweight.

SPSS Statistics for Windows version 28.0.0 (IBM Corp., Armonk, NY, USA) was used for data analysis and a significance level of 0.05 was chosen. We estimated mean differences in motor scores between the VLBW and control groups using linear regression with motor scores as the dependent variable, group as the fixed factor, and cohort, age, and sex as covariates. Differences in SD were calculated as the mean difference divided by the SD of the control group. Normality of residuals was evaluated by visual inspection of Q–Q plots. Bootstrapping was applied with *B* = 2000 samples and a bias‐corrected and accelerated method owing to deviations from normality for all motor variables except the BMAT Short Form item ‘grip strength’ and the Revised HiMAT items ‘walk over obstacle’, ‘skip’, and ‘hop forward’. We performed sensitivity analyses by excluding participants with NSI, since NSI is known to affect motor abilities. In supplementary analyses, we also adjusted for maternal age, and paternal and maternal education, as possible confounders. These variables were entered as covariates in the regression analyses. We adjusted overall and fine motor abilities for handedness as it may act as an ancestor of outcome. We also explored the role of height as a mediator on the association between VLBW and overall and gross motor abilities (Figure 1) using the PROCESS macro for IBM SPSS (www.processmacro.org) as developed by Hayes.

A priori power calculations suggested on the basis of previous follow‐up numbers (total VLBW *n* = 170, control *n* = 200) that we would have power to detect differences of 0.29 SD with an alpha‐level of 0.05 and a power of 80%, and 0.40 SD with an alpha‐level of 0.01 and 90% power.

## RESULTS

### Overall motor abilities

**FIGURE 2:**
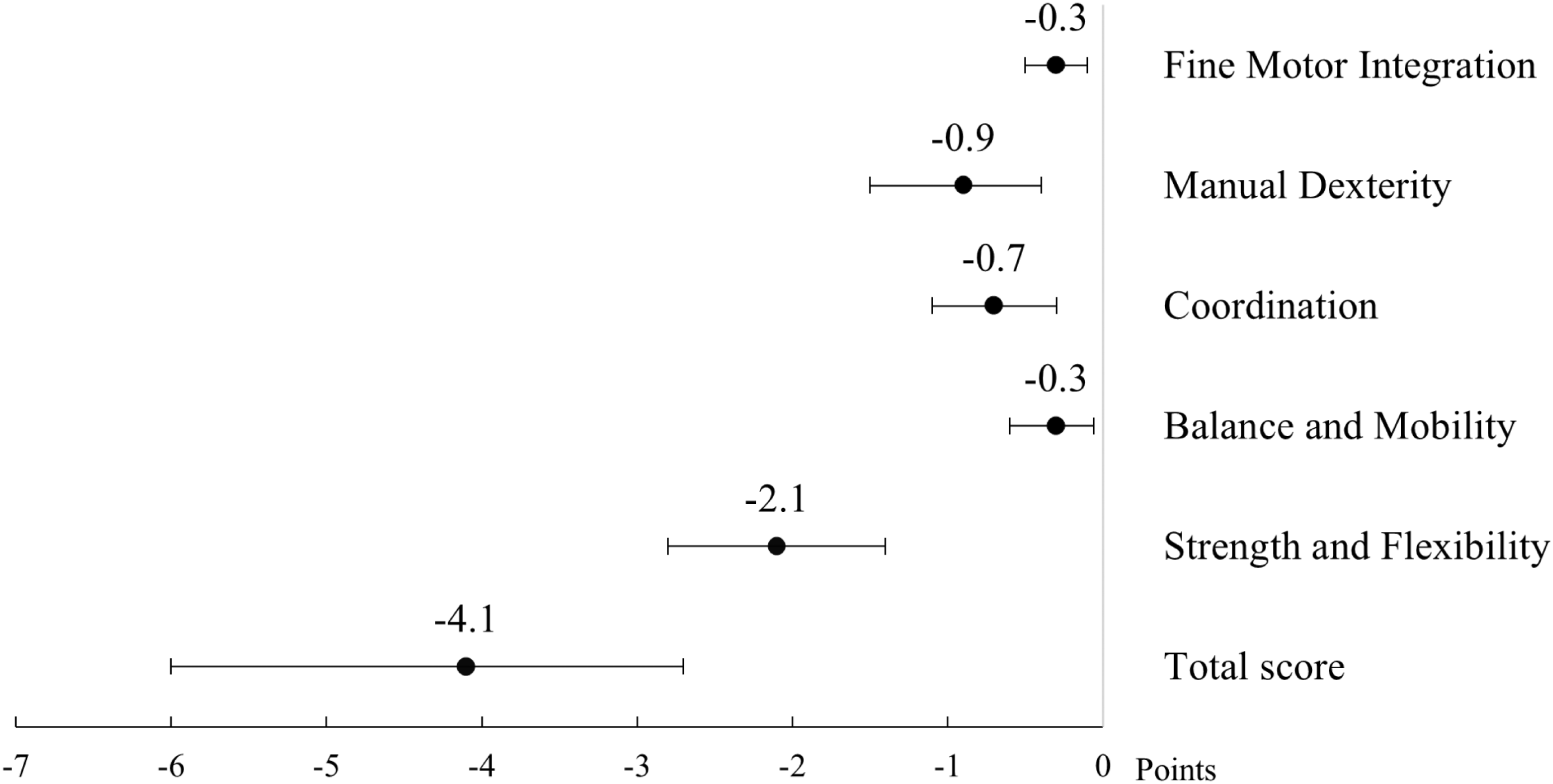
Mean differences in Bruininks Motor Ability Test Short Form scores between the VLBW and the control groups adjusted for cohort, age, and sex. Horizontal lines indicate 95% confidence intervals, based on bias‐corrected and accelerated bootstrap. Abbreviation: VLBW, very low birthweight.

BMAT Short Form total score and subtest scores are shown in Figure 2 and raw scores in Table 3. The mean total score was 61.1 (SD 8.8) points in the VLBW group and 65.3 (SD 3.1) points in the control group (mean difference − 4.1, 95% confidence interval [CI] −6.0 to −2.7), corresponding to a difference of −1.32 SD. The VLBW group had lower raw and subtest scores than the control group on all subtests, except for the Balance and Mobility subtest.

**TABLE 3:** Mean differences in Bruininks Motor Ability Test Short Form scores between the VLBW and control groups adjusted for cohort, age, and sex.

	VLBW			Control			Mean difference adjusted for cohort, age, and sex (95% CI)^a^	
	*n*	Mean	(SD)	*n*	Mean	(SD)		
Fine Motor Integration								
Drawing a Line through a Path – Curved (*n* errors)	118	0.1	(0.4)	147	0.0	(0.0)	0.1	(0.04–0.2)
Marking Shapes (*n* seconds)	118	8.6	(5.2)	147	6.9	(2.1)	1.6	(0.8–2.5)
Manual Dexterity								
Transferring Pennies – Preferred Hand (*n* pennies in 15 seconds)	117	16.2	(2.6)	146	17.1	(1.5)	−0.8	(−1.3 to −0.3)
Stringing With Blocks in Nonpreferred Hand (*n* blocks in 15 seconds)	116	7.2	(1.3)	147	7.6	(1.0)	−0.4	(−0.7 to −0.1)
Coordination								
Dropping and Catching a Ball – Both Hands (*n* catches)	115	4.9	(0.3)	147	5.0	(0.1)	−0.1	(−0.1 to −0.01)
Catching a Tossed Ball – One Hand (*n* catches)	115	4.1	(1.3)	146	4.6	(0.9)	−0.4	(−0.7 to −0.2)
Balance and Mobility								
Standing on One Leg on a Line – Eyes Open (*n* seconds up to 10 seconds)	114	9.8	(1.2)	147	10.0	(0.0)	−0.2	(−0.4 to 0.0)
Walking Alternating Directions (*n* seconds)	117	14.0	(4.2)	147	12.4	(1.7)	1.6	(1.0–2.2)
Strength and Flexibility								
Grip Strength – Nonpreferred Hand (*n* closures in 30 seconds)	115	33.3	(15.5)	147	42.1	(12.8)	−8.4	(−12.1 to −4.9)
Wall Push‐ups (*n* push‐ups in 30 seconds)	116	18.4	(5.1)	146	21.6	(4.4)	−3.1	(−4.2 to −1.9)

^a^ Based on bias‐corrected and accelerated bootstrap.

Abbreviations: CI, confidence interval; SD, standard deviation; VLBW, very low birthweight.

### Fine motor abilities

The VLBW group spent more time (in seconds) to complete the GP and the TMT‐5 (Table 4). Differences in SD ranged from 0.72 to 0.93 for the GP and was 0.33 for the TMT‐5.

**TABLE 4:** Mean differences in Grooved Pegboard and Trail Making Test‐5 scores between the VLBW and control groups adjusted for cohort, age, and sex.

	VLBW			Control			Mean difference adjusted for cohort, age, and sex (95% CI)^a^	
	*n*	Mean	(SD)	*n*	Mean	(SD)		
Grooved Pegboard – dominant hand								
Raw score (seconds)	116	64.2	(15.2)	146	58.4	(7.8)	5.6	(2.7–9.0)
Grooved Pegboard – non‐dominant hand								
Raw score (seconds)	114	72.6	(20.0)	147	63.6	(9.4)	8.7	(5.1–12.8)
Trail Making Test‐5								
Raw score (seconds)	117	23.3	(10.2)	146	20.9	(7.0)	2.4	(0.3–4.5)

^a^ Based on bias‐corrected and accelerated bootstrap.

Abbreviations: CI, confidence interval; SD, standard deviation; VLBW, very low birthweight.

### Gross motor abilities

**FIGURE 3:**
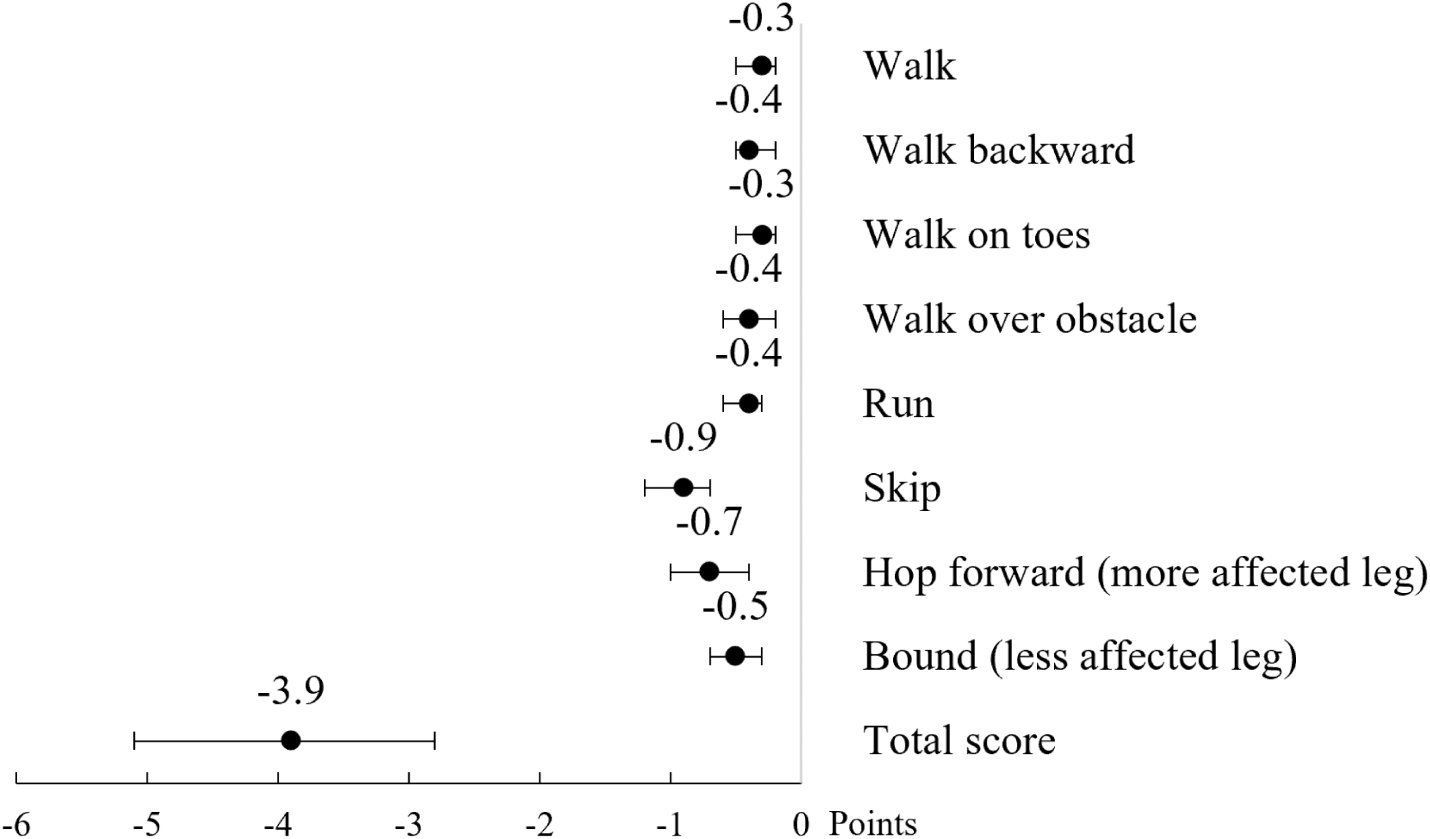
Mean differences in Revised High‐level Mobility Assessment Tool scores between the VLBW and the control groups adjusted for cohort, age, and sex. Horizontal lines indicate 95% confidence intervals, based on bias‐corrected and accelerated bootstrap. Abbreviation: VLBW, very low birthweight.

Revised HiMAT total and item scores are shown in Figure 3 and raw scores in Table 5. Mean total score was 23.0 (SD 5.8) points in the VLBW group and 27.2 (SD 4.2) points in the control group (mean difference − 3.9, 95% CI −5.1 to −2.8), corresponding to −0.93 SD. The VLBW group had lower scores on all eight items.

**TABLE 5:** Mean differences in Revised High‐level Mobility Assessment Tool scores between the VLBW and control groups adjusted for cohort, age, and sex.

	VLBW			Control			Mean difference adjusted for cohort, age, and sex (95% CI)^a^	
	*n*	Mean	(SD)	*n*	Mean	(SD)		
Walk (seconds)	114	4.4	(0.8)	147	3.9	(0.7)	0.5	(0.3–0.7)
Walk backward (seconds)	114	6.2	(2.1)	147	5.1	(1.2)	1.1	(0.7–1.6)
Walk on toes (seconds)	113	5.2	(1.2)	146	4.6	(1.0)	0.6	(0.3–0.9)
Walk over obstacle (seconds)	114	4.6	(0.9)	147	4.0	(0.8)	0.6	(0.4–0.8)
Run (seconds)	112	2.1	(0.4)	146	1.9	(0.3)	0.2	(0.1–0.3)
Skip (seconds)	102	4.0	(1.5)	143	3.2	(0.7)	0.8	(0.5–1.1)
Hop forward (more affected leg) (seconds)	102	5.5	(2.1)	137	4.4	(1.5)	1.1	(0.6–1.5)
Bound (less affected leg) (cm)	110	122.4	(25.2)	147	138.1	(24.3)	−15.4	(−20.7 to −10.2)

^a^ Based on bias‐corrected and accelerated bootstrap.

Abbreviations: CI, confidence interval; SD, standard deviation; VLBW, very low birthweight.

### Sensitivity analyses

When we excluded participants with NSI, BMAT Short Form total score and subtest scores and Revised HiMAT total score were reduced but still significant except for the BMAT Short Form subtests Manual Dexterity and Balance and Mobility (Figures S2 and S3). There were no longer differences on the BMAT Short Form items ‘drawing a line through a path’, ‘transferring pennies’, and ‘standing on one leg on a line’ (Table S1). Furthermore, mean difference in TMT‐5 raw score was reduced to 1.2 seconds (95% CI −0.6 to 2.9), and no longer significant.

### Supplementary analyses

When we adjusted overall and fine motor abilities for handedness, the mean difference between the groups barely changed (data not shown). Adjustment for maternal age, and maternal and paternal education, did not change any of the results (data not shown). The indirect effect of height as a mediator on the association between VLBW and total scores of the BMAT Short Form and Revised HiMAT was −1.3 (95% CI −2.0 to −0.7) and − 0.6 (95% CI −1.1 to −0.2) respectively (Tables S2 and S3). The indirect effect of height was also significant for all BMAT Short Form subtest scores except Manual Dexterity and all Revised HiMAT item scores except the ‘walk backward’, ‘skip’, and ‘hop forward’ item scores.

## DISCUSSION

This is the first study to report clinically assessed motor abilities in adults born preterm with VLBW beyond the age of 23 years. We found that adults born with VLBW had lower scores on the BMAT Short Form than control individuals. They also had lower scores on the other fine (GP and TMT‐5) and gross (Revised HiMAT) motor tests. Differences in overall and gross motor abilities were partly mediated by height. When we excluded participants with NSI, mean differences were reduced but most remained significant.

A strength of this study is the comprehensive joint assessment of two Nordic birth cohorts with harmonized methods. The HeSVA participants were born between 1978 and 1985 and the NTNU LBW Life participants between 1986 and 1988. We consider the temporality effects to be small between the 1970s and 1980s as all participants were born before the surfactant era in the 1990s. In addition, we adjusted for cohort in our analyses.

Loss to follow‐up is unavoidable in cohort studies and may lead to selection bias and reduced statistical power.^19^ About 51% of the invited individuals were assessed. The considerable number who did not participate may be partly explained by the data collection occurring during the COVID‐19 pandemic, making participants unable to attend in person.

Individuals with VLBW who did not consent to participation or who did not have their motor abilities assessed seemed to have a higher prevalence of NSI (18.9%) than those who participated (8.5%). This may indicate that the better‐functioning participants met for assessment, which may have resulted in underestimated group differences, which is often the case with attrition bias.^20^ Even though a limited sample may reduce statistical power, we still found significant differences between the groups.

Trained examiners were blinded to group adherence and previous test results, and procedures were audited to ensure consistency across examiners within and between study sites. We performed motor assessments using standardized motor tests designed for adults. The BMAT Short Form is designed to give a reliable and valid measure of overall adult motor abilities which can be used in research.^14^ With lack of validated assessment tools for adults of this age, we chose the BMAT Short Form as the primary outcome measure even though it is recommended for ages 40 years and older.^14^ The GP is a commonly used test to measure fine motor abilities.^15^ The TMT‐5 specifically measures motor speed and is part of a larger test battery to evaluate higher‐level cognitive function in children, adolescents, and adults.^16^ The Revised HiMAT provides a measure of gross motor abilities and has high interrater reliability, retest reliability, and internal consistency.^18^ Although it was initially developed to measure mobility limitations after traumatic brain injury, it can be applied to other neurological conditions. As the tools used in this study are mainly designed to detect motor difficuties in impaired populations, this could have led to insensitivity in detecting subtle motor difficulties. Thus, our findings of poorer overall, fine, and gross motor scores between adults born with VLBW and control individuals suggest that adults born with VLBW have substantial difficulties in motor abilities.

The only previous study with participants of a similar age showed increased self‐reported motor coordination difficulties in adults with extremely low birthweight aged 29 to 36 years.^8^ Motor coordination was assessed by a single item, ‘I am poorly coordinated/clumsy’. We were able to confirm these findings using a battery of standardized motor tests in a slightly larger sample.

Adults born with VLBW had poorer overall motor abilities than the control group, reflected by a mean difference corresponding to −1.32 SD on the BMAT Short Form. Although there is lack of evidence from adult populations, the size of our difference is in line with reviews and meta‐analyses of children and adolescents. Evensen et al.^1^ reported differences between control individuals and VLBW children, adolescents, and young adults with a typical magnitude of 1.0 SD on overall motor abilities measured by different motor assessment tools, while de Kieviet et al.^3^ reported that children born very preterm/VLBW performed 0.51 to 0.94 SD poorer than control individuals on the Bruininks–Oseretsky Test of Motor Proficiency, the child version of BMAT. When we excluded participants with NSI, there were no significant differences between the groups on the Manual Dexterity and Balance and Mobility subtests of the BMAT Short Form. The means of both groups were close to the maximum score, indicating a ceiling effect.^14^

As hypothesized, our study indicated reduced motor speed in fine motor tests, with mean differences corresponding to 0.72 and 0.93 SD on the GP, and 0.33 SD on the TMT‐5. In adolescence, we found larger differences on the GP in the NTNU LBW Life cohort, corresponding to 1.84 and 1.47 SD,^21^ whereas in young adulthood the differences corresponded to 0.89 and 1.16 SD.^7^ On the TMT‐5 the differences corresponded to 1.55 SD, substantially larger than our findings. Furthermore, our findings are well in line with those of de Kieviet et al.^3^ who reported a mean difference of 0.62 SD in manual dexterity on the Movement Assessment Battery for Children.

We also found reduced motor speed in gross motor tasks. The Revised HiMAT consists mostly of timed items and the results were robust, even after excluding participants with NSI. A change of 2 points reflects a clinically important change.^17^ Thus, our finding of a mean difference of −3.9 points corresponding to −0.93 SD is likely to be clinically significant. Our results are in line with findings of the NTNU LBW Life cohort at 23 years, where the VLBW group had 3.3 points lower score than the control group, corresponding to a difference of −1.32 SD.^7^

The poorer motor abilities with reduced motor speed in the VLBW group could possibly be explained by brain pathology observed in individuals born preterm. Magnetic resonance imaging studies from the NTNU LBW Life cohort found structural changes in the brain of VLBW children, adolescents, and young adults.^11^ These changes have been shown to correlate with poor motor skills in the same cohort persisting from childhood^22^, ^23^, ^24^ through adolescence^21^, ^25^, ^26^ and up to young adulthood.^27^

Vision may also play a role in reduced motor abilities. Sripada et al.^28^ reported an increased risk of visual–motor integration deficits in young adults born preterm with VLBW, and reduced scores on fine motor tests correlated with alterations in white matter tracts and reduced surface area of visual–perceptual cortical regions. This could be of special importance for the GP and TMT‐5, which require use of complex visual–motor coordination.^15^, ^16^ Visual deficits, especially visual acuity, were found to influence motor problems in adolescents with VLBW.^29^

Motor difficulties have been associated with decreased social self‐concept in adults born very preterm/VLBW at age 26 years.^5^ Reduced self‐concept together with poor motor abilities might interfere with the ability to participate both in social and in physical activities.^1^ Poor motor abilities may also affect daily living activities and academic achievement,^6^ which in our study was reflected by fewer adults born with VLBW having attained higher‐level education than control individuals. The lower educational levels could also be explained by decreased cognitive functioning which has been observed in adults born with VLBW,^30^ and this may again be related to the brain‐ and visual pathology discussed.

Enhanced knowledge of poor adult motor abilities among individuals born preterm with VLBW may thus create a better understanding and awareness among healthcare professionals to provide appropriate intervention, promote participation, and reduce impact on everyday activities.^1^

In conclusion, our findings of poorer overall, fine, and gross motor abilities among adults born preterm with VLBW compared with term‐born individuals indicate that those born preterm with VLBW have substantial difficulties in motor function that last into mid‐adulthood.

## FUNDING INFORMATION

The follow‐up study was funded by the Joint Research Committee of St. Olavs Hospital HF and the Faculty of Medicine and Health Sciences (46055600‐159), Norwegian University of Science and Technology (NTNU). The work of EK and KAIE was supported by the European Union's Horizon 2020 Research and Innovation Program: Research on European Children and Adults Born Preterm (RECAP Preterm) Consortium Preterm Project (733280). SDB received funding from the Dam Foundation (2021/FO347467) and the Central Norway Regional Health Authority (90654402). KADA received funding from the Department of Clinical and Molecular Medicine, NTNU. APMJ received funding from the Faculty of Medicine and Health Sciences, NTNU. LJ received funding from University of Oulu Graduate School, University of Oulu, Signe and Ane Gyllenberg Foundation, and The Alma and KA Snellman Foundation, Oulu, Finland. MK received funding from The Eye and Tissue Bank Foundation, The Eye Foundation, Evald and Hilda Nissi Foundation, and Mary and Georg C. Ehrnrooth Foundation. EK received funding from the Academy of Finland (315690), the Novo Nordisk Foundation (NNF20OC0063930), the Foundation for Pediatric Research, the Sigrid Juselius Foundation, The Finnish Medical Foundation, the Finnish Foundation for Cardiovascular Research, and the Finnish Diabetes Research Foundation. KAIE received funding from the Joint Research Committee of St. Olavs Hospital HF and the Faculty of Medicine and Health Sciences, NTNU (30283).

## Supporting information


**Figure S1:** Flowchart of participants.

**Figure S2:** Mean differences in Bruininks Motor Ability Test Short Form scores between the VLBW group and the control group adjusted for cohort, age, and sex when participants with neurosensory impairment were excluded.

**Figure S3:** Mean differences in Revised High‐level Mobility Assessment Tool scores between the VLBW group and the control group adjusted for cohort, age, and sex when participants with neurosensory impairment were excluded.

**Table S1:** Mean differences in motor scores between the VLBW group and the control group adjusted for cohort, age, and sex when participants with neurosensory impairment were excluded.

**Table S2:** Direct, indirect, and total effect of VLBW on Bruininks Motor Ability Test Short Form scores with height as mediator.

**Table S3:** Direct, indirect, and total effect of VLBW on Revised High‐level Mobility Assessment Tool scores with height as mediator.

## Data Availability

The datasets generated during and/or analyzed during the current study are not publicly available because permission has not been applied from either the participants or the Ethical Committee; but aggregated data may be available from the corresponding author upon reasonable request.
