# Supplementary material for: Motor abilities in adults born with very low birthweight: A study of two birth cohorts from Finland and Norway
Source: Dev Med Child Neurol. 2024 Feb 18;66(9):1190–200. doi: 10.1111/dmcn.15883 (PMC11579805; doi:10.1111/dmcn.15883)
Supplement: Supplementary file 1 — Figure S1: Flowchart of participants. [file DMCN-66-1190-s001.docx]

**
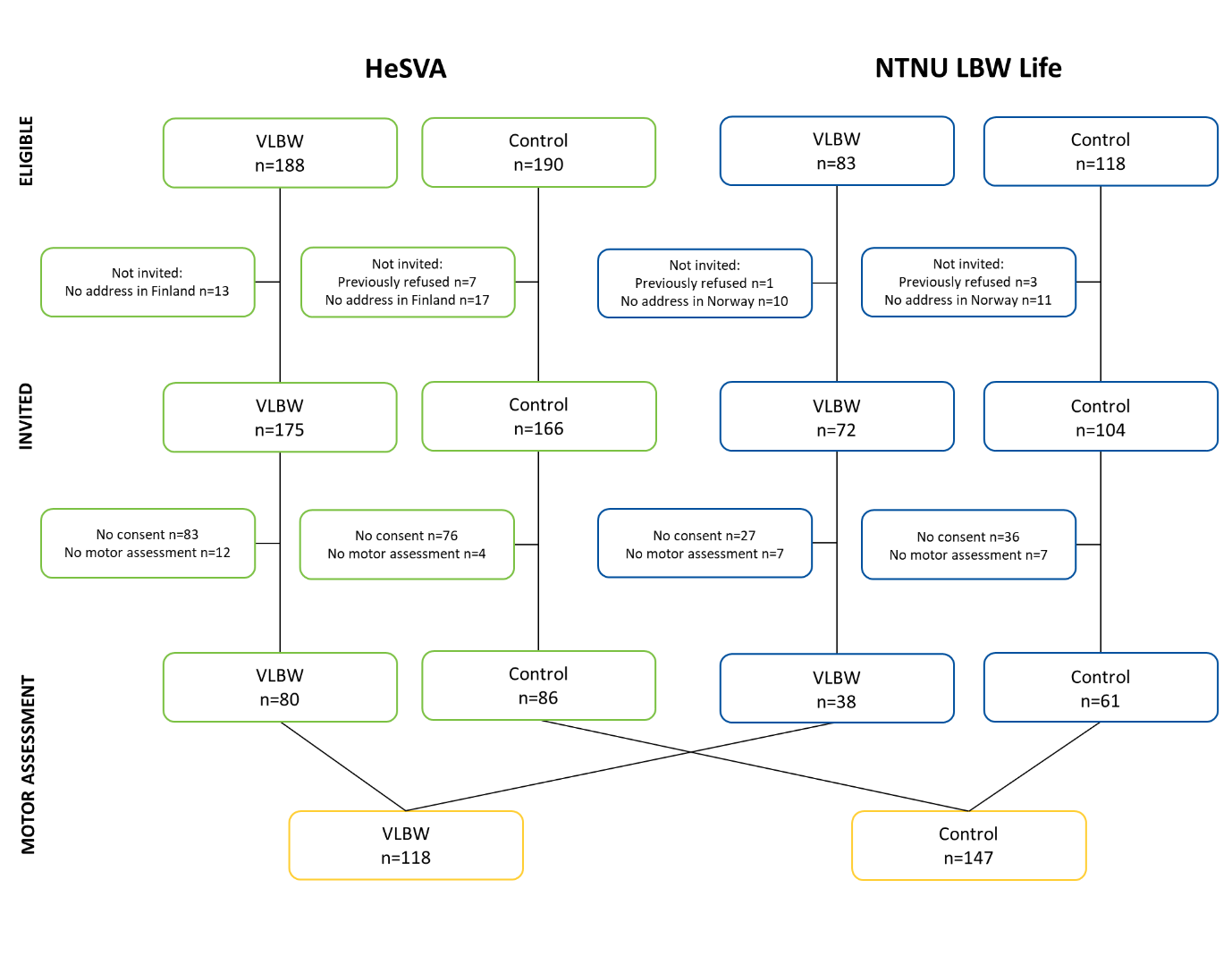
**

**Figure S1:** Flowchart of participants.

Abbreviations: HeSVA, Helsinki Study of Very Low Birth Weight Adults; NTNU LBW Life, NTNU Low Birth Weight in a Lifetime Perspective; VLBW, very low birth weight.
