# Supplementary material for: Motor abilities in adults born with very low birthweight: A study of two birth cohorts from Finland and Norway
Source: Dev Med Child Neurol. 2024 Feb 18;66(9):1190–200. doi: 10.1111/dmcn.15883 (PMC11579805; doi:10.1111/dmcn.15883)
Supplement: Supplementary file 2 — Figure S2: Mean differences in Bruininks Motor Ability Test Short Form scores between the VLBW group and the control group adjusted for cohort, age, and sex when participants with neurosensory impairment were excluded. [file DMCN-66-1190-s003.docx]

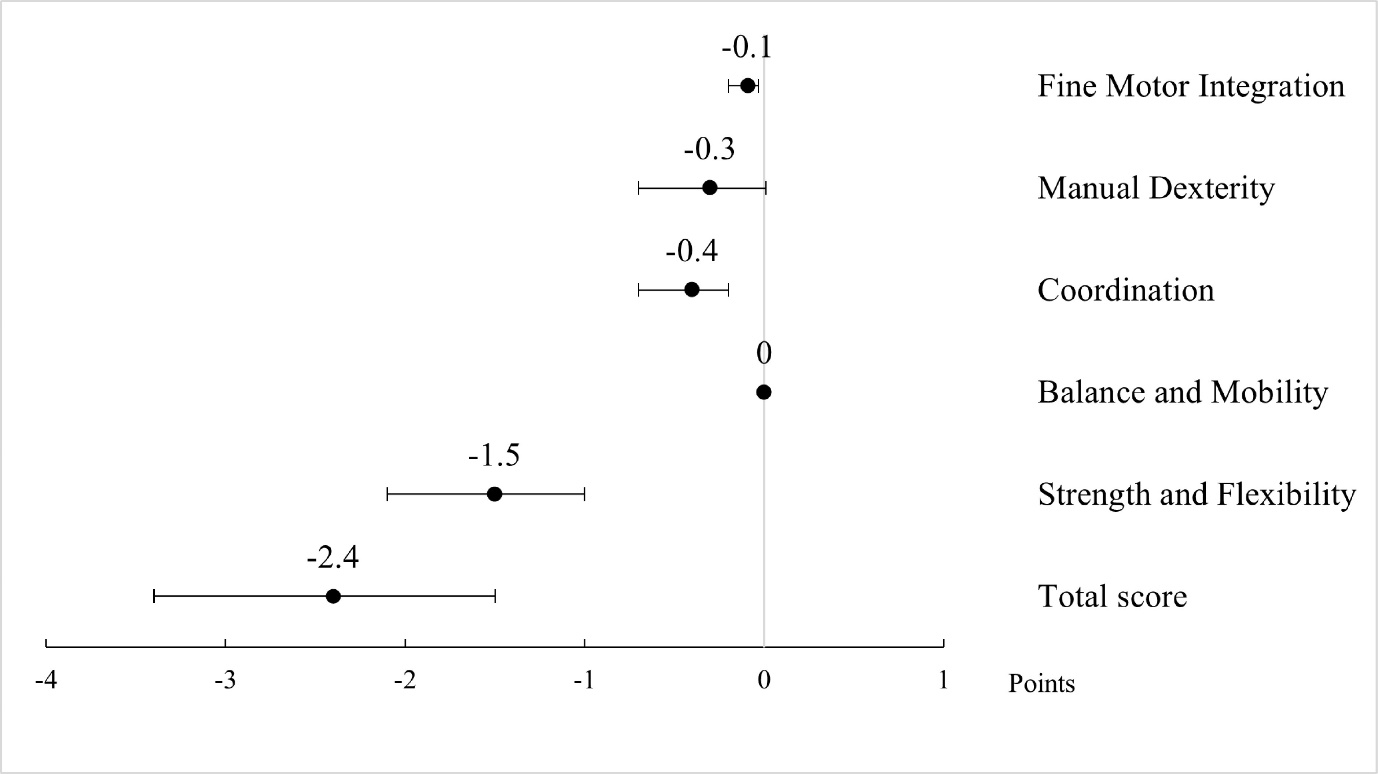


**Figure S2:** Mean differences in Bruininks Motor Ability Test Short Form scores between the VLBW group and the control group adjusted for cohort, age, and sex when participants with neurosensory impairment were excluded.

Horizontal lines indicate 95% confidence intervals, based on bias-corrected and accelerated bootstrap.

Abbreviation: VLBW, very low birth weight.
