# Supplementary material for: Motor abilities in adults born with very low birthweight: A study of two birth cohorts from Finland and Norway
Source: Dev Med Child Neurol. 2024 Feb 18;66(9):1190–200. doi: 10.1111/dmcn.15883 (PMC11579805; doi:10.1111/dmcn.15883)
Supplement: Supplementary file 3 — Figure S3: Mean differences in Revised High‐level Mobility Assessment Tool scores between the VLBW group and the control group adjusted for cohort, age, and sex when participants with neurosensory impairment were excluded. [file DMCN-66-1190-s004.docx]

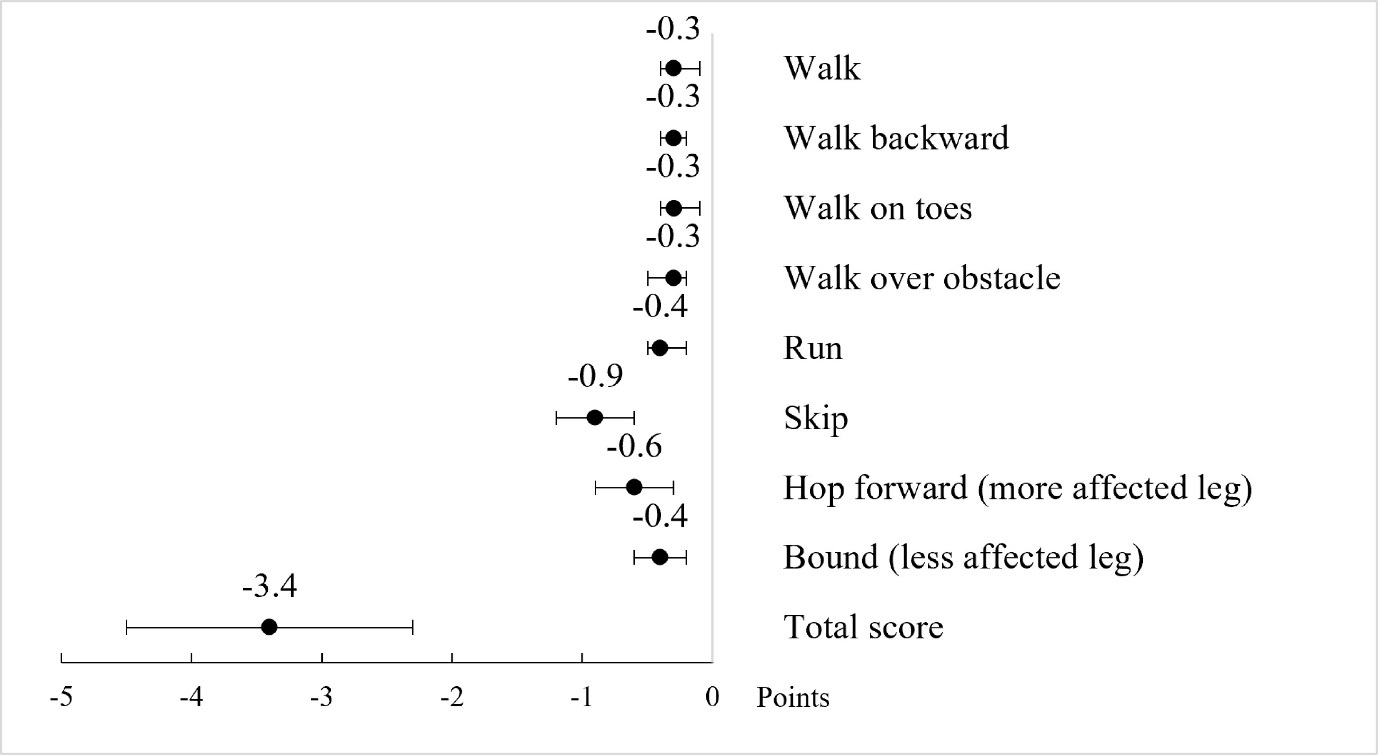


**Figure S3:** Mean differences in Revised High-level Mobility Assessment Tool scores between the VLBW group and the control group adjusted for cohort, age, and sex when participants with neurosensory impairment were excluded.

Horizontal lines indicate 95% confidence intervals, based on bias-corrected and accelerated bootstrap.

Abbreviation: VLBW, very low birth weight.
