# Supplementary material for: Motor abilities in adults born with very low birthweight: A study of two birth cohorts from Finland and Norway
Source: Dev Med Child Neurol. 2024 Feb 18;66(9):1190–200. doi: 10.1111/dmcn.15883 (PMC11579805; doi:10.1111/dmcn.15883)
Supplement: Supplementary file 4 — Table S1: Mean differences in motor scores between the VLBW group and the control group adjusted for cohort, age, and sex when participants with neurosensory impairment were excluded. [file DMCN-66-1190-s005.docx]

**Table S1:** Mean differences in motor scores between the VLBW group and the control group adjusted for cohort, age and sex when participants with neurosensory impairment were excluded.

|  | **VLBW** | | | **Control** | | | **Mean difference adjusted for cohort, age and sex (95% CI)^a^** | |
| --- | --- | --- | --- | --- | --- | --- | --- | --- |
|  | ***n*** | **Mean** | **(SD)** | ***n*** | **Mean** | **(SD)** |  |  |
| **Bruininks Motor Ability Test Short Form** |  |  |  |  |  |  |  |  |
| *Fine Motor Integration* |  |  |  |  |  |  |  |  |
| Drawing a Line Through a Path – Curved (*n* errors) | 108 | 0.1 | (0.3) | 146 | 0.0 | (0.0) | 0.1 | (0.0-0.1) |
| Marking Shapes (*n* seconds) | 108 | 7.8 | (2.6) | 146 | 6.9 | (2.1) | 0.8 | (0.2-1.3) |
| *Manual Dexterity* |  |  |  |  |  |  |  |  |
| Transferring Pennies – Preferred Hand (*n* pennies in 15 seconds) | 108 | 16.6 | (1.9) | 145 | 17.1 | (1.5) | -0.4 | (-0.7 to 0.02) |
| Stringing With Blocks in Nonpreferred Hand (*n* blocks in 15 seconds) | 108 | 7.4 | (1.1) | 146 | 7.6 | (1.0) | -0.3 | (-0.5 to -0.004) |
| *Coordination* |  |  |  |  |  |  |  |  |
| Dropping and Catching a Ball – Both Hands (*n* catches) | 108 | 4.9 | (0.3) | 146 | 5.0 | (0.1) | -0.1 | (-0.1 to -0.01) |
| Catching a Tossed Ball – One Hand (*n* catches) | 108 | 4.2 | (1.2) | 145 | 4.6 | (0.8) | -0.4 | (-0.7 to -0.1) |
| *Balance and Mobility* |  |  |  |  |  |  |  |  |
| Standing on One Leg on a Line – Eyes Open (*n* seconds up to 10 seconds) | 108 | 10.0 | (0.0) | 146 | 10.0 | (0.0) | 0.0 | (0.0-0.0) |
| Walking Alternating Directions (*n* seconds) | 108 | 13.3 | (2.1) | 146 | 12.4 | (1.7) | 1.0 | (0.5-1.5) |
| *Strength and Flexibility* |  |  |  |  |  |  |  |  |
| Grip Strength – Nonpreferred Hand (*n* closures in 30 seconds) | 108 | 33.9 | (15.5) | 146 | 42.3 | (12.8) | -8.0 | (-11.4 to -4.3) |
| Wall Push-ups (*n* push-ups in 30 seconds) | 108 | 18.9 | (4.7) | 145 | 21.6 | (4.4) | -2.6 | (-3.8 to -1.4) |
| **Grooved Pegboard and Trail Making Test-5** |  |  |  |  |  |  |  |  |
| Grooved Pegboard – dominant hand (seconds) | 107 | 62.2 | (10.5) | 145 | 58.2 | (7.5) | 3.6 | (1.4-6.0) |
| Grooved Pegboard – non-dominant hand (seconds) | 107 | 70.5 | (15.9) | 146 | 63.3 | (9.0) | 6.6 | (3.7-9.8) |
| Trail Making Test-5 (seconds) | 108 | 22.0 | (6.7) | 145 | 20.8 | (7.0) | 1.2 | (-0.6 to 2.9) |
| **Revised High-level Mobility Assessment Tool** |  |  |  |  |  |  |  |  |
| Walk (seconds) | 108 | 4.4 | (0.7) | 146 | 3.9 | (0.7) | 0.4 | (0.3-0.6) |
| Walk backward (seconds) | 108 | 5.9 | (1.4) | 146 | 5.0 | (1.1) | 0.8 | (0.5-1.1) |
| Walk on toes (seconds) | 107 | 5.1 | (1.1) | 145 | 4.6 | (0.9) | 0.5 | (0.3-0.8) |
| Walk over obstacle (seconds) | 108 | 4.5 | (0.8) | 146 | 4.0 | (0.8) | 0.5 | (0.3-0.7) |
| Run (seconds) | 106 | 2.1 | (0.4) | 145 | 1.9 | (0.3) | 0.2 | (0.1-0.2) |
| Skip (seconds) | 99 | 3.9 | (1.3) | 142 | 3.2 | (0.6) | 0.7 | (0.4-1.0) |
| Hop forward (more affected leg) (seconds) | 100 | 5.4 | (2.1) | 137 | 4.4 | (1.5) | 1.0 | (0.6-1.5) |
| Bound (less affected leg) (cm) | 105 | 124.7 | (22.7) | 146 | 138.4 | (24.1) | -13.3 | (-18.5 to -8.3) |

^a^Based on bias-corrected and accelerated bootstrap.

Abbreviations: CI, confidence interval; SD, standard deviation; VLBW, very low birth weight.
