# Supplementary material for: Motor abilities in adults born with very low birthweight: A study of two birth cohorts from Finland and Norway
Source: Dev Med Child Neurol. 2024 Feb 18;66(9):1190–200. doi: 10.1111/dmcn.15883 (PMC11579805; doi:10.1111/dmcn.15883)
Supplement: Supplementary file 5 — Table S2: Direct, indirect, and total effect of VLBW on Bruininks Motor Ability Test Short Form scores with height as mediator. [file DMCN-66-1190-s002.docx]

**Table S2:** Direct, indirect and total effect of VLBW on Bruininks Motor Ability Test Short Form scores with height as mediator.

|  | **Direct effect of VLBW** | | **Indirect effect of VLBW** | | **Total effect of VLBW** | |
| --- | --- | --- | --- | --- | --- | --- |
|  | **Estimate** | **(95% CI)** | **Estimate** | **(95% CI)** | **Estimate** | **(95% CI)** |
| Total Score | -2.8 | (-4.4 to -1.2) | -1.3 | (-2.0 to -0.7) | -4.1 | (-5.7 to -2.6) |
| Fine Motor Integration | -0.2 | (-0.4 to -0.04) | -0.05 | (-0.1 to -0.002) | -0.3 | (-0.4 to -0.1) |
| Manual Dexterity | -0.8 | (-1.3 to -0.2) | -0.1 | (-0.3 to 0.1) | -0.9 | (-1.4 to -0.4) |
| Coordination | -0.4 | (-0.8 to -0.1) | -0.3 | (-0.4 to -0.1) | -0.7 | (-1.0 to -0.3) |
| Balance and Mobility | -0.1 | (-0.3 to 0.1) | -0.1 | (-0.4 to -0.003) | -0.3 | (-0.5 to -0.1) |
| Strength and Flexibility | -1.3 | (-2.0 to -0.6) | -0.7 | (-1.1 to -0.4) | -2.0 | (-2.7 to -1.4) |

Analyses adjusted for cohort, age and sex.

Abbreviations: CI, confidence interval; VLBW, very low birth weight.
