# Supplementary material for: Motor abilities in adults born with very low birthweight: A study of two birth cohorts from Finland and Norway
Source: Dev Med Child Neurol. 2024 Feb 18;66(9):1190–200. doi: 10.1111/dmcn.15883 (PMC11579805; doi:10.1111/dmcn.15883)
Supplement: Supplementary file 6 — Table S3: Direct, indirect, and total effect of VLBW on Revised High‐level Mobility Assessment Tool scores with height as mediator. [file DMCN-66-1190-s006.docx]

**Table S3:** Direct, indirect and total effect of VLBW on Revised High-level Mobility Assessment Tool scores with height as mediator.

|  | **Direct effect of VLBW** | | **Indirect effect of VLBW** | | **Total effect of VLBW** | |
| --- | --- | --- | --- | --- | --- | --- |
|  | **Estimate** | **(95% CI)** | **Estimate** | **(95% CI)** | **Estimate** | **(95% CI)** |
| Total score | -3.4 | (-4.5 to -2.2) | -0.6 | (-1.1 to -0.2) | -3.9 | (-5.0 to -2.8) |
| Walk | -0.2 | (-0.4 to -0.1) | -0.1 | (-0.1 to -0.02) | -0.3 | (-0.5 to -0.2) |
| Walk backward | -0.3 | (-0.5 to -0.2) | -0.1 | (-0.1 to 0.001) | -0.4 | (-0.5 to -0.2) |
| Walk on toes | -0.2 | (-0.4 to -0.1) | -0.1 | (-0.1 to -0.01) | -0.3 | (-0.5 to -0.2) |
| Walk over obstacle | -0.3 | (-0.5 to -0.2) | -0.1 | (-0.1 to -0.01) | -0.4 | (-0.6 to -0.2) |
| Run | -0.4 | (-0.5 to -0.2) | -0.1 | (-0.1 to -0.004) | -0.4 | (-0.6 to -0.3) |
| Skip | -0.8 | (-1.1 to -0.5) | -0.1 | (-0.3 to 0.03) | -0.9 | (-1.2 to -0.7) |
| Hop forward (more affected leg) | -0.7 | (-1.0 to -0.4) | -0.01 | (-0.1 to 0.1) | -0.7 | (-1.0 to -0.4) |
| Bound (less affected leg) | -0.4 | (-0.6 to -0.2) | -0.1 | (-0.2 to -0.1) | -0.5 | (-0.7 to -0.3) |

Analyses adjusted for cohort, age and sex.

Abbreviations: CI, confidence interval; VLBW, very low birth weight.
